# Silicon enhanced salt tolerance by improving the root water uptake and decreasing the ion toxicity in cucumber

**DOI:** 10.3389/fpls.2015.00759

**Published:** 2015-09-17

**Authors:** Shiwen Wang, Peng Liu, Daoqian Chen, Lina Yin, Hongbing Li, Xiping Deng

**Affiliations:** ^1^State Key Laboratory of Soil Erosion and Dryland Farming on the Loess Plateau, Institute of Soil and Water Conservation, Northwest A&F UniversityYangling, China; ^2^Institute of Soil and Water Conservation, Chinese Academy of SciencesYangling, China; ^3^Department of Plant Protection, Shandong Agricultural UniversityTai’an, China; ^4^College of Life Sciences, Northwest A&F UniversityYangling, China

**Keywords:** silicon, salt tolerance, root hydraulic conductance, polyamine, water balance

## Abstract

Although the effects of silicon application on enhancing plant salt tolerance have been widely investigated, the underlying mechanism has remained unclear. In this study, seedlings of cucumber, a medium silicon accumulator plant, grown in 0.83 mM silicon solution for 2 weeks were exposed to 65 mM NaCl solution for another 1 week. The dry weight and shoot/root ratio were reduced by salt stress, but silicon application significantly alleviated these decreases. The chlorophyll concentration, net photosynthetic rate, transpiration rate and leaf water content were higher in plants treated with silicon than in untreated plants under salt stress conditions. Further investigation showed that salt stress decreased root hydraulic conductance (Lp), but that silicon application moderated this salt-induced decrease in Lp. The higher Lp in silicon-treated plants may account for the superior plant water balance. Moreover, silicon application significantly decreased Na^+^ concentration in the leaves while increasing K^+^ concentration. Simultaneously, both free and conjugated types of polyamines were maintained at high levels in silicon-treated plants, suggesting that polyamines may be involved in the ion toxicity. Our results indicate that silicon enhances the salt tolerance of cucumber through improving plant water balance by increasing the Lp and reducing Na^+^ content by increasing polyamine accumulation.

## Introduction

Salt stress has a detrimental effect on plant growth, resulting in decreased crop production (Hasegawa et al., 2000; Zhu, 2003; Munns and Tester, 2008). To reduce the damaging effects of salt stress on plant production, a variety of methods are currently applied. Of these, silicon application is a promising method (Guntzer et al., 2012). Silicon, the second most abundant element in the earth’s crust, has been found to alleviate salt stress in various ways (Epstein, 1999, 2009). The beneficial effect of silicon on salt stress has been investigated in different plant species, such as sorghum, rice, wheat, tomato, barley, and others (Liang et al., 2003; Romero-Aranda et al., 2006; Saqib et al., 2008; Zhu and Gong, 2014; Liu et al., 2015). The mechanisms thus far identified by previous studies as being involved in silicon-induced salt tolerance include reducing Na^+^ uptake by inhibiting bypass flow or restricting transpiration (Matoh et al., 1986; Gong et al., 2006), improving leaf water content (Romero-Aranda et al., 2006), increasing antioxidant enzyme activity (Zhu et al., 2004), and increasing plasma membrane H^+^-ATPase activity (Liang et al., 2006). The fact that so many different complex biological functions have been identified in different studies suggests that the mechanisms by which silicon improves plant salt tolerance have not been well established.

Salt typically stresses plants in two ways, namely, through osmotic stress and through ion toxicity. High concentrations of salt in solution result in osmotic stress, which limits water availability in plants and affects water balance. Ion toxicity is a result of salt accumulation to toxic concentrations in old leaves, which accelerates the senescence of old, leaves and leads to leaf death. As osmotic stress and ion toxicity are the predominant effects of salt stress, plants have correspondingly adapted to salt stress by decreasing their susceptibility to these effects under salt stress conditions (Munns and Tester, 2008). Most previous studies have focused on how silicon decreases Na^+^ accumulation in the shoots and maintains ion homeostasis (Yin et al., 2015). The prevailing consensus on how silicon decreases Na^+^ accumulation is based on a theory in which silicon functions as a “mechanical barrier,” directly or indirectly decreasing Na^+^ uptake. For example, silicon reduces transpiration through deposition in the cell walls of leaves, leading to decreases in Na^+^ uptake and transpiration volume (Matoh et al., 1986); in rice, likewise, silicon is deposited in the roots where it reduces Na^+^ uptake by reducing bypass flow (Gong et al., 2006). Recently, Yin et al. (2015) reported that silicon reduced Na^+^ uptake and accumulation by mediating some important metabolic processes related to ion chancel regulation, and reported for the first time that polyamine metabolism participates in this regulation.

Compared with the effect of silicon on ion toxicity regulation, the effect of silicon on alleviating salt-induced osmotic stress and the mechanism underlying this effect have been largely ignored (Liu et al., 2015). High concentrations of salt in solution result in increased osmotic stress, which limits water absorption by the plant and in turn affects leaf water content, stomatal conductance (gs), leaf growth and photosynthesis (Boursiac et al., 2005; Munns and Tester, 2008). Chen et al. (2014) clearly proved that silicon enhances salt tolerance by alleviating salt-induced osmotic stress, which plays a more important role than salt-induced ion toxicity. Romero-Aranda et al. (2006) demonstrated that silicon improved tomato salt tolerance through enhancing leaf water content. In addition, several studies have reported that plants treated with silicon maintain a higher stomatal conductance and transpiration rate (Gong et al., 2006; Farshidi et al., 2012; Yin et al., 2013). These results suggest that silicon application can improve plant water balance under salt-induced osmotic stress conditions. Recently, Liu et al. (2015) reported that enhanced root hydraulic conductance achieved through aquaporin regulation accounts for silicon’s alleviating effect on salt-induced osmotic stress in sorghum.

Although previous studies have illustrated the mechanisms by which silicon alleviates salt stress through mitigating both ion toxicity and osmotic stress in certain plant species, these mechanisms need further and deeper study. Firstly, most previous studies on silicon and salt stress have discussed either ion toxicity or osmotic stress, overlooking the possible effects of the integration of these two effects in a single plant species. Secondly, the content of silicon in plant materials varies greatly, from over 10% to below 0.05% (Epstein, 1999). In rice, a species with a high capacity for silicon uptake and accumulation, it has recently been confirmed that silicon works as a mechanical barrier, decreasing Na^+^ uptake and transport to the shoot; yet it should not be assumed that this mechanical barrier effect operates in the same way in other plants, especially in plants with lower capacities for silicon uptake and accumulation. Determining whether other mechanisms are also involved will require further study. Yin et al. (2015) reported that polyamines are involved in regulating ion homeostasis and thus in enhancing sorghum salt tolerance, but more investigation will be necessary to determine whether the same mechanism exists in other plants. Thirdly, silicon has been reported to decrease salt-induced osmotic stress by enhancing root hydraulic conductance, but this has been confirmed only in sorghum under short-term salt stress (24 h). The role of silicon in decreasing osmotic stress needs further study in more plant species. Therefore, the mechanisms by which silicon treatment alleviates salt stress need further study, especially in plant species that accumulate much lower levels of silicon.

Cucumber is a medium silicon accumulator plant: its silicon content is far lower than that of rice or sorghum (Ma and Yamaji, 2006), for example. Cucumber seedlings are sensitive to salt stress, which can be alleviated by silicon application (Zhu et al., 2004; Liu et al., 2014). It is also a good plant material for measuring root hydraulic conductance (Lee et al., 2004). In this study, taking the results of previous studies into consideration, we hypothesized that (1) silicon enhanced cucumber salt tolerance both by improving the water balance and decreasing the ion toxicity; (2) silicon improved the plant water balance by enhancing the root water uptake ability; (3) silicon alleviated the ion toxicity by accumulating polyamines, which are involved in regulating ion homeostasis. In order to test these hypotheses, the biomass, gas exchange, water contents, root hydraulic conductance, ion concentration and polyamine levels were investigated in cucumber seedlings treated with silicon and salt.

## Materials and Methods

### Seedling Cultivation, Silicon and Salt Treatment

Cucumber (*Cucumis sativus* L. cv. Jin chun 10) seeds were sterilized with 10% NaClO_4_ and germinated in filter paper for 24 h. The seeds were then sown in vermiculite for 1 week. After the cotyledons expanded, the seedlings were transplanted into plastic containers each containing 20 L of 1/2 Hoagland solution (pH 6.0). Three days after transplantation, half of the seedlings were supplied with 0.8 mM H_2_SiO_3_. H_2_SiO_3_ was produced by passing sodium silicate solution through a column filled with cation-exchange resin (Yin et al., 2013). NaCl was added into the hydroponic culture on the night of the sixth day at a concentration of 65 mM. The seedlings were sampled after 1.5, 3.5, and 7.5 days of salt treatment. During the experiment, the culture solution was aerated and exchanged every 3 days. The pH of the culture solution was adjusted to 6.0 by the addition of 0.1 M HCl and 1 M KOH every day. Each seedling in the experiment was subjected to one of four possible treatments: control, silicon, NaCl, and NaCl + silicon. All experiments were conducted in a growth chamber which was set to a 14/10 h day/night cycle at a day/night temperature of 28/20°C with 40–50% relative humidity. The amount of photosynthetically active radiation (PAR) received by the upper plant surfaces was 500 mol m^-2^ s^-1^.

### Fresh Weight and Root/Shoot Ratio

After 1.5, 3.5, and 7.5 days of salt treatment, the leaf, stem, and root were sampled individually and weighed immediately. The total weight and root/shoot ratio were calculated. Three replications were performed under each condition (each container was considered one replication), and each replication included five plants.

### Chlorophyll Concentration, Net Photosynthetic Rate and Transpiration Rate

Fully expanded upper leaves were used for measuring the chlorophyll concentration. Each sample of 0.5 g fresh leaf was extracted with 80% acetone on a shaker at room temperature until the tissue was totally bleached. The chlorophyll was measured and calculated according to the method of Arnon (1949). Net photosynthetic rate and transpiration rate were measured with a portable photosynthesis system (Li-6400; LI-COR Inc., Lincoln, NE, USA) between 10:00 AM and 12:00 PM. The leaf was placed in a 6 cm^2^ chamber at a photo flux density of 500 μmol m^-2^s^-1^. Three replications were performed under each condition (each container was considered one replication and each replication included five plants).

### Water Content and Leaf and Root Osmotic Potential

After 1.5, 3.5, and 7.5 days of salt treatment, the leaves were sampled and their fresh weights (FWs) were measured immediately. After deactivation of enzymes at 105°C for 30 min, the samples were finally dried at 70°C for 3 days to enable calculation of the dry weight (DW). The water content was calculated according to the following equation: Water content = (FW-DW)/FW × 100%. For osmotic potential analysis, frozen leaf or root (0.5 g) samples were first placed in 0.5 mL tubes and allowed to thaw at room temperature. After thawing, 0.5 mL tubes were drilled at the bottom, placed into 1.5 mL tubes, and centrifuged at 4000 rpm for 5 min to gather the cell sap. The osmolarity of the collected sap was determined using a dew point microvolt meter (Model 5600, Wescor, Logan, UT, USA), and the osmotic potential was calculated. Three replications were performed under each condition (each container was considered one replication and each replication included five plants).

### Root Hydraulic Conductance

Root hydraulic conductance (Lp) measurements were carried out as described by Liu et al. (2015). After 7.5 days of treatment, each shoot was removed at the base of the root system leaving a 4-cm stem. The root was inserted into a pressure chamber filled with the corresponding solution, then sealed with silicon seals. The pressure was increased in steps of 0.1 MPa up to 0.3 MPa (P_gas_). The exuded sap from the stem was collected with absorbent cotton and weighed. For a given pressure, the volume exuded from the root system was plotted against time. The slopes of these relationships referred to the unit root dry mass, which yields the volume flow, J_vr_ (mg g^-1^ min^-1^). Root hydraulic conductance was calculated as Lp = J_vr_/P_gas_. Three replications were performed under each condition (each container was considered one replication and each replication included two plants).

### Ion Content

Dried leaf, stem and root samples were milled to powder, weighed, and then digested by nitric acid in a bottle tube at 320°C for 5 h. Na^+^ and K^+^ concentration were measured by atomic adsorption spectrometer with a flame photometer (ZL5100, PerkinElmer Inc., USA). Three replications were performed under each condition (each container was considered one replication and each replication included five plants).

### Polyamine Quantification

Root tips, each 0.5 g (0–1 cm), were ground and homogenized in 5 mL of 5% HClO_4_ and extracted on a shaker at room temperature for 24 h. After centrifuging, the extracted supernatant was used for measuring free-type polyamines. The residues of plant extracts were washed with 5% HClO_4_, then subjected to hydrolysis in 6 M HCl at 110°C for 15 h. The filtrated hydrolyzates were allowed to evaporate to dryness, and the residues were dissolved in 5% HClO_4_ for the measurement of conjugated-type polyamines. The polyamine concentration was analyzed by high-performance liquid chromatography (HPLC: LC-10A, Shimadzu, Kyoto, Japan) according to Yin et al. (2014). Three replications were performed under each condition (each container was considered one replication and each replication included five plants).

### Statistical Analysis

Data were subjected to analysis of variance (ANOVA) using the Statistical Analysis System (SAS version 8.0) software. The differences between the means were compared using the Tukey-Kramer test (*p* < 0.05). Four independent experiments were conducted in most portions of this study, including biomass and photosynthetic rate, transpiration rate, leaf water content and ion concentration. Osmotic potential and root hydraulic conductance were measured three times; polyamine was measured twice.

## Results

### Silicon Enhanced Cucumber Salt Tolerance

**FIGURE 1:**
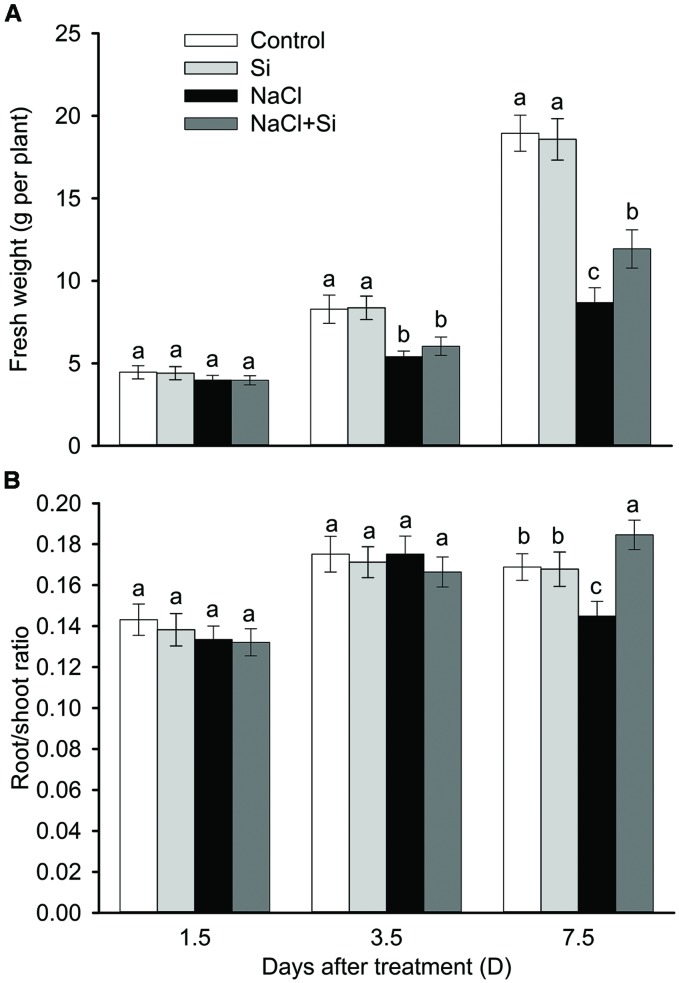
**Effects of silicon (0.83 mM) and salt stress (65 mM) on the fresh weight **(A)** and root/shoot ratio **(B)** of cucumber seedlings**. All parameters were measured after 1.5, 3.5, and 7.5 days of salt treatment. Vertical bars represent the standard deviations (*n* = 3). Different letters at the same time point represent significant differences (*p* < 0.05) between the treatments.

**FIGURE 2:**
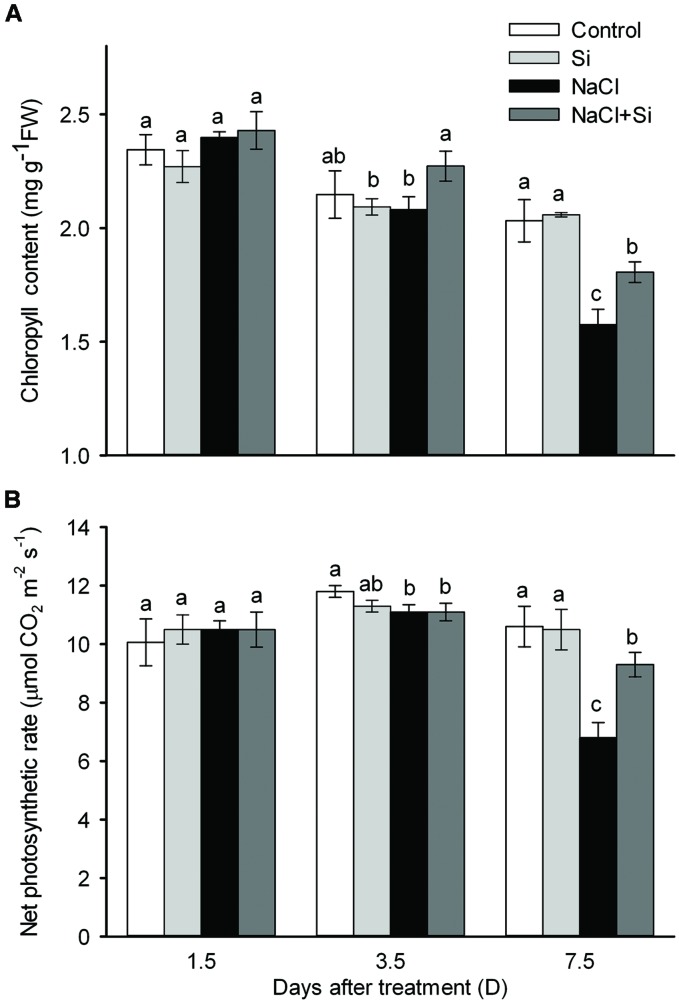
**Effects of silicon (0.83 mM) and salt stress (65 mM) on leaf chlorophyll concentration **(A)** and net photosynthetic rate (B).** All parameters were measured after 1.5, 3.5, and 7.5 days of salt treatment. Vertical bars represent the standard deviations (*n* = 3). Different letters at the same time point represent significant differences (*p* < 0.05) between the treatments. Sharing the same letter indicates no significant difference between the treatments.

As shown in **Figure 1A**, no effect of silicon on biomass was observed under control conditions. Salt stress significantly decreased plant growth after 3.5 and 7.5 days of treatment. Silicon application clearly alleviated the salt-induced inhibition of growth. After 7.5 days salt treatment, the FW of silicon-treated plants was 37% higher than that of untreated plants. In addition, the root/shoot ratio was decreased by salt stress after 7.5 days of treatment, and silicon application moderated this decrease (**Figure 1B**). Chlorophyll concentration was not affected by salt or silicon after 1.5 and 3.5 days of salt treatment. After 7.5 days of salt treatment, however, chlorophyll concentration was significantly decreased; silicon application alleviated this salt-induced decrease in chlorophyll concentration. Similarly, the net photosynthetic rate was decreased after 7.5 days of salt treatment, and silicon application significantly alleviated this decrease (**Figure 2**). These results show that silicon application enhanced cucumber salt tolerance.

### Silicon Alleviated Plant Water Balance Under Salt Stress

**FIGURE 3:**
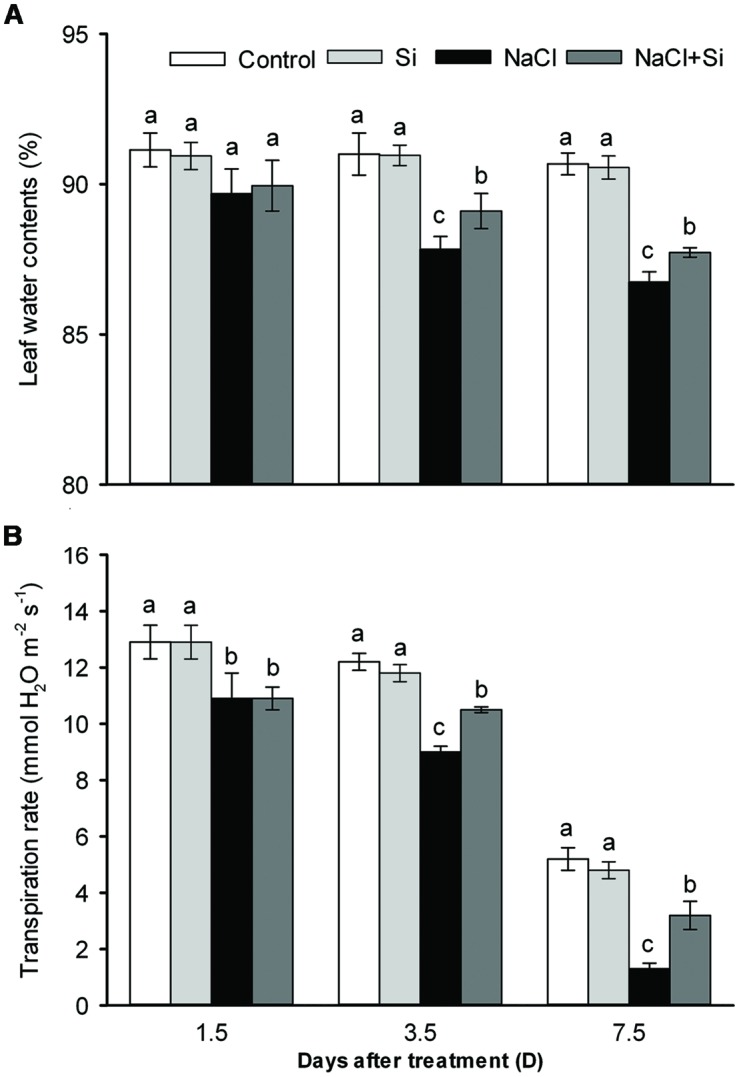
**Effects of silicon (0.83 mM) and salt stress (65 mM) on leaf water content **(A)** and transpiration rate (B).** All parameters were measured after 1.5, 3.5, and 7.5 days of salt treatment. Vertical bars represent the standard deviations (*n* = 3). Different letters at the same time point represent significant differences (*p* < 0.05) between the treatments.

**FIGURE 4:**
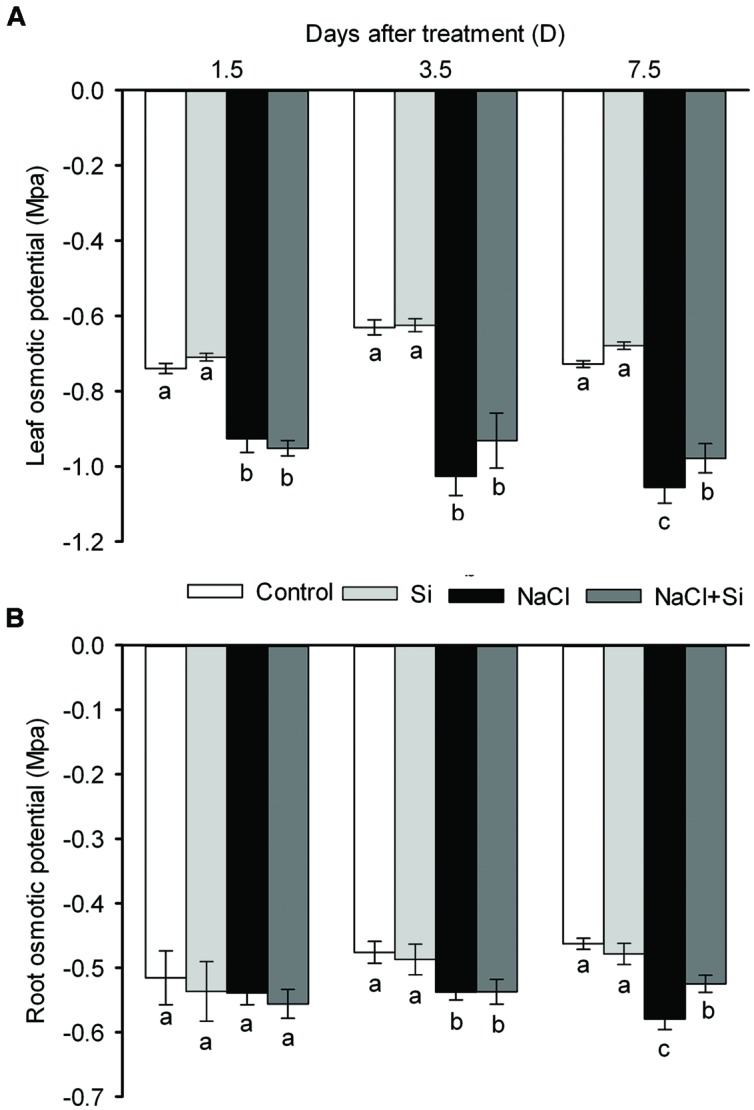
**Effects of silicon (0.83 mM) and salt stress (65 mM) on leaf **(A)** and root osmotic potential (B).** All parameters were measured after 1.5, 3.5, and 7.5 days of salt treatment. Vertical bars represent the standard deviations (*n* = 3). Different letters at the same time point represent significant differences (*p* < 0.05) between the treatments.

Leaf water content was decreased by salt stress. After 1.5 days of treatment, silicon application did not affect the leaf water content; after longer treatment periods, however, silicon significantly reduced the salt-induced decrease in leaf water content (**Figure 3A**). Transpiration rate was likewise decreased by salt stress. After short-term (1.5 days) salt treatment, silicon application had no effect on transpiration rate; after 3.5 and 7.5 days of salt treatment, however, silicon application obviously alleviated the salt-induced decrease in transpiration rate (**Figure 3B**). Osmotic potential, as shown in **Figure 4**, was decreased by salt stress in both leaves and roots, but silicon application enabled both plant tissue types to maintain their osmotic potential at higher levels than untreated plants did. These results show that silicon can alleviate salt-induced plant water imbalance.

### Silicon Moderated the Salt-Induced Decrease in Root Hydraulic Conductance

**FIGURE 5:**
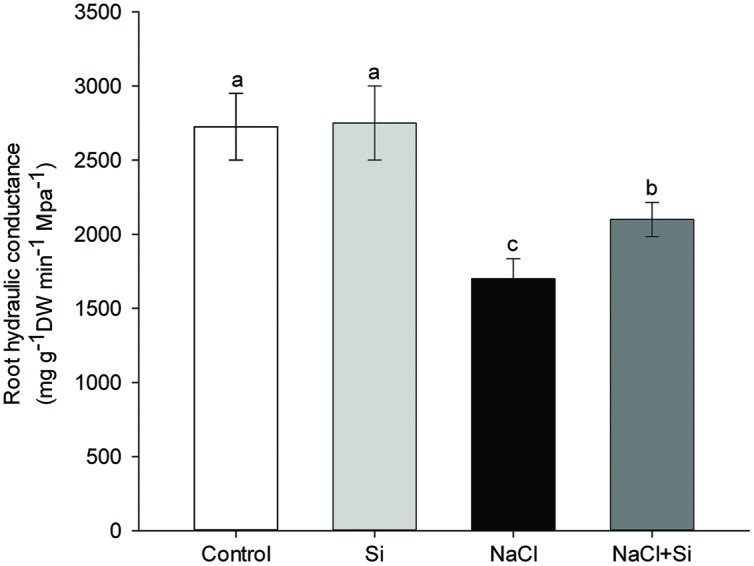
**Effects of silicon (0.83 mM) and salt stress (65 mM) on root hydraulic conductance (Lp).** The Lp was measured after 7.5 days of salt treatment. Vertical bars represent the standard deviations (*n* = 3). Different letters represent significant differences (*p* <0.05) between the treatments.

Under normal growth conditions, silicon application does not change root hydraulic conductance. Salt stress, on the other hand, dramatically decreases root hydraulic conductance, and, under salt stress conditions, silicon application significantly reverses this decrease. Root hydraulic conductance under salt stress was 24% higher in silicon-treated plants compared to untreated plants (**Figure 5**). These results show that plants treated with silicon had a greater capacity for root water uptake under salt stress compared to untreated plants.

### Silicon Application Decreased Leaf Na Toxicity

**FIGURE 6:**
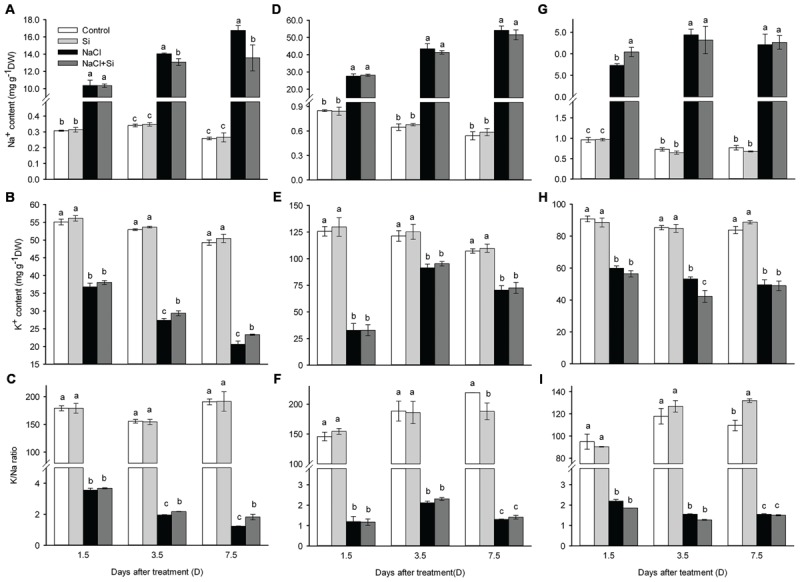
**Effects of silicon (0.83 mM) and salt stress (65 mM) on Na^+^ (**A**, leaf; **D**, stem; **G**, root), K^+^ (**B**, leaf; **E**, stem; **H**, root) concentration and K^+^/Na^+^ ratio (**C**, leaf; **F**, stem; **I**, root).** All parameters were measured after 1.5, 3.5, and 7.5 days of salt treatment. Vertical bars represent the standard deviations (*n* = 3). Different letters at the same time point represent significant differences (*p* < 0.05) between the treatments.

As shown in **Figure 6**, the Na^+^ concentration was sharply increased under salt stress in leaves, stems and roots. In stems and roots, silicon application did not decrease the Na^+^ concentration after 1.5, 3.5, and 7.5 days of salt treatment. In leaves, however, the Na^+^ concentration was maintained at lower levels in silicon-treated plants than in untreated plants after 3.5 and 7.5 days of salt stress. Salt stress significantly decreased the K^+^ concentration both with and without silicon. The K^+^ concentration in stems and roots was not affected by silicon under either normal or salt stress conditions. In leaves, however, the K^+^ concentration was higher in plants treated with silicon than in untreated plants. Likewise, in stems and roots, the K/Na ratio was not affected by silicon, whereas in leaves the K/Na ratio was maintained at a higher level in silicon-treated plants than in untreated plants after 3.5 and 7.5 days of treatment (**Figure 6C**).

### Silicon Induced Polyamine Accumulation Under Salt Stress

**FIGURE 7:**
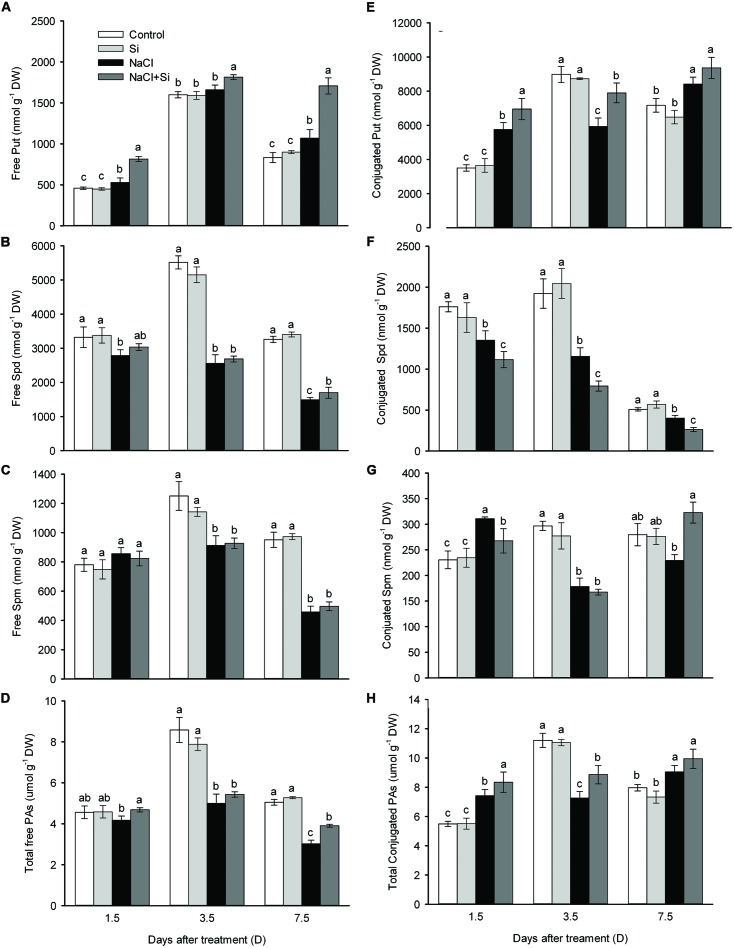
**Effects of silicon (0.83 mM) and salt stress (65 mM) on polyamines (PAs) concentrations (putrescine, Put; spermidine, Spd; and spermine, Spm; **(A)** Free Put; **(B)** Free Spd; **(C)** Free Spm; **(D)** Total free polyamines; **(E)** Conjugated Put; **(F)** Conjugated Spd; **(G)** Conjugated Spm; **(H)** Total conjugated polyamines)**. Root tips (1 cm) were used for measurement. All parameters were measured after 1.5, 3.5, and 7.5 days of salt treatment. Vertical bars represent the standard deviations (*n* = 3). Different letters at the same time point represent significant differences (*p* < 0.05) between the results of the treatments. Sharing the same letter indicates no significant difference.

In roots, the total free polyamines were not affected by silicon application under normal conditions. Salt stress decreased the total free polyamines after 3.5 and 7.5 days, but silicon application moderated this salt-induced decrease in polyamine levels. Three free polyamines were considered separately. Salt stress decreased the putrescine concentration, while silicon application reversed this decrease. Salt stress also decreased the spermidine concentration, while silicon application alleviated this decrease only after 7.5 days of treatment. Salt stress also decreased the free spermine concentration, but silicon did not affect this decrease under either normal or salt stress conditions. As for conjugated-type polyamines, silicon application enhanced the putrescine and spermine levels, but caused a decrease in spermidine concentration (**Figure 7**).

## Discussion

### Silicon Enhanced Cucumber Salt Tolerance by Ameliorating Leaf Water Balance and Decreasing Ion Toxicity

Salt stresses plants by inducing osmotic stress and ion toxicity, and plants adapt to salt stress by decreasing salt-induced osmotic stress and ion toxicity (Munns and Tester, 2008). In the present study, silicon application significantly alleviated the salt-induced biomass reduction (**Figure 1**), indicating that silicon application enhances cucumber salt tolerance. High concentrations of salts outside the roots result in increased osmotic stress, which induces root water uptake difficulties, causing leaf water imbalance and ultimately a reduction in plant growth (Boursiac et al., 2005; Munns and Tester, 2008). In the present study, silicon application alleviated the salt-induced decreases in leaf water content and transpiration rate (**Figure 2**), indicating that silicon application ameliorate the leaf water balance and alleviates salt-induced osmotic stress. Therefore, the amelioration of water balance by silicon application seems to contribute to silicon’s enhancement of plant salt tolerance.

Ion toxicity is considered a major factor in the disturbance of regular plant growth that occurs under salt stress (Zhu, 2003). Ion toxicity is a result of salt accumulation to toxic concentrations in the leaves, which decreases chlorophyll concentrations and accelerates leaf senescence, leading to decreased photosynthetic rate and leaf death (Katerji et al., 2003; Munns and Tester, 2008; Liu and Shi, 2010). A growing body of evidence has demonstrated that, under salt stress, silicon application can reduce sodium concentrations (Ashraf et al., 2010; Zhu and Gong, 2014; Yin et al., 2015). In the present study, Na^+^ concentrations in the stems and roots were not affected by silicon application, but Na^+^ concentration in the leaves was decreased by silicon application. In addition, K^+^ concentration and K^+^/Na^+^ ratio in the leaves were enhanced by silicon application (**Figure 6**). Correspondingly, chlorophyll concentration in the leaves was maintained at a higher level in silicon-treated plants than in untreated plants (**Figure 2**), which further supports the conclusion that silicon decreased the salt-induced ion toxicity.

### Silicon-Induced Amelioration of Leaf Water Balance Related with Enhances Root Hydraulic Conductance and Root/Shoot Ratio

Plants have certain strategies for maintaining water balance when they are exposed to osmotic stress (Horie et al., 2011). Osmotic adjustment helps plants to retain water despite low water potential, and thus to alleviate osmotic stress; it has been proposed that osmotic adjustment is the mechanism by which silicon treatment contributes to maintaining water balance in tomato plants under salt stress (Romero-Aranda et al., 2006). In the present study, leaf and root osmotic potentials were not decreased by silicon (**Figure 4**). This result demonstrates that osmotic adjustment cannot be the mechanism by which silicon helps to maintain water balance in cucumber. Another mechanism by which plants can regulate water balance is modulation of the transpiration rate. When plants are exposed to osmotic stress, their immediate response is to close the stomata to decrease the transpiration rate and thereby to reduce water loss (Cornic, 2000). The closure of the stomata also reduces CO_2_ fixation and decreases the photosynthetic rate. In order for plant growth to continue, however, the plant must maintain an optimal stomata aperture. Transpiration rates are much higher in salt-tolerant species than in salt-sensitive species (e.g., maize and sorghum) under salt stress conditions (Nagy et al., 1995). In the present study, the transpiration rate was decreased by salt stress; yet silicon application alleviated this decrease in transpiration rate (**Figure 3B**). Thus silicon-treated plants maintained high transpiration rates and leaf water contents, as did salt-tolerant species. This implies that silicon’s effect on plant water balance is achieved through increasing water uptake rather than reducing water loss.

Root is the primary site for plants to uptake water. Root hydraulic conductance represents water uptake capacity, and mainly depends on the driving force, root anatomy, and root water permeability (Steudle, 2000; Sutka et al., 2011). Osmotic stress is the first stress experienced when a plant is exposed to saline soil; it has an immediate influence on plant growth (Horie et al., 2011). One of the primary responses of plants to osmotic stress is a decrease in root hydraulic conductance (Lp) (Boursiac et al., 2005). In the present study, the salt-induced decrease in Lp was significantly moderated by silicon application (**Figure 5**), suggesting that silicon application enhanced plant water uptake under salt stress. The same result was obtained by Liu et al. (2015) in sorghum, proving that silicon enhances root hydraulic conductance under short-term salt stress (24 h). Upon long-term (>3 days) exposure to drought stress, roots can respond with changes in root surface and anatomical structures, which in turn cause profound changes in the plant’s water uptake (Javot and Maurel, 2002). In this study, the root/shoot ratio was increased by silicon after 7.5 days of treatment (**Figure 1B**), suggesting that silicon-mediated modifications of root morphology may also account for the increased water uptake ability of silicon-treated plants.

### Promotion of Polyamine Accumulation Could be Involved in Silicon-Induced Decrease in Ion Toxicity

Ion toxicity is considered as the major factor that inhibits the plant growth under salt stress. The fact that silicon application reduces sodium accumulation has been confirmed in various studies (Ashraf et al., 2010; Zhu and Gong, 2014; Yin et al., 2015). In the present study, silicon decreased the Na^+^ and increased the K^+^ concentration in the leaves. Several possible mechanisms by which silicon may decrease leaf Na^+^ concentrations have been proposed in previous studies. Matoh et al. (1986) proposed that the decrease in Na^+^ concentration in rice was due to a silicon-induced decrease in transpiration rate. In the present study, however, the transpiration rate was actually increased by silicon. Gong et al. (2006) proposed that silicon deposited in the exodermis and endodermis of the rice root caused an impairment of the apoplastic Na^+^ transport pathway in the roots. These suggestions were based on the hypothesis that plants can uptake large quantities of silicon and generate a physical/mechanical barrier through silicon accumulation. It is likely, however, that this is not the main mechanism responsible for the effects observed in the current study, because cucumber is a medium silicon-accumulator while rice is a high silicon-accumulator. The silicon concentration in rice was much higher than that in cucumber (Epstein, 1999).

How did silicon reduce the Na^+^ concentration in cucumber? Recently, Yin et al. (2015) reported that the accumulation of polyamines induced by silicon treatment may be involved in regulating the ion homeostasis in sorghum under short-term salt stress conditions. Polyamine was found to block plasma membranes K^+^ and non-selective cation channels, which assisted in the retention of intracellular K^+^ and the reduction of Na^+^ influx, thus ameliorating the detrimental effects of salt stress on plant ionic homeostasis (Zhao et al., 2007; Zepeda-Jazo et al., 2011; Velarde-Buendía et al., 2012; Yin et al., 2015). In this study, under salt stress, silicon induced polyamine accumulation and the K/Na homeostasis was alleviated (**Figures 6** and **7**). This observation is in keeping with that of Yin et al. (2015) in sorghum, suggesting that polyamine may be involved in silicon’s reduction of ion toxicity in this study.

### Silicon Could Actively Regulate Plant Water Balance and Ion Homeostasis

Considering all results of the present study, it is supposed that silicon application alleviated the adverse effects of salt on cucumber growth through maintaining plant water balance and decreasing ion toxicity. Silicon enhanced root hydraulic conductance and the root/shoot ratio, which enhanced root water uptake ability, improving plant water balance. Silicon also induced polyamine accumulation, which contributed to maintaining ion homeostasis and reducing salt-induced ion toxicity. This study supports the hypothesis than silicon alleviates salt stress by alleviating salt-induced the osmotic stress and ion toxicity simultaneously. Although the deeper mechanisms are not well understood, the present study suggests that silicon does not play a mechanical role in salt tolerance in cucumber. Along with the results of previous studies in sorghum (Liu et al., 2015; Yin et al., 2015), the present results show that silicon is involved in regulating water balance in medium silicon-accumulator plants as well as abundant silicon-accumulator plants. Therefore, silicon enhances root hydraulic conductance and affects polyamine metabolism in cucumber, suggesting that silicon could actively mediate some important metabolic processes that enhance stress tolerance in various silicon accumulator plants.

## Author Contributions

SW planned and conducted the experiment, collected and analyzed the data, and prepared the draft. PL, DC helped measurements of polyamines contents. LY, HL, and XD helped in drafting the manuscript and interpretation the results.

## Conflict of Interest Statement

The authors declare that the research was conducted in the absence of any commercial or financial relationships that could be construed as a potential conflict of interest.
